# O-GlcNAcylation determines the function of the key O-GalNAc glycosyltransferase C1GalT1 in bladder cancer: O-GlcNAcylation governs pro-tumorigenic role of C1GalT1 in bladder cancer

**DOI:** 10.3724/abbs.2024129

**Published:** 2024-08-08

**Authors:** Yazhuo Jiang, Jinpeng Wu, Feng Guan, Liang Liang, Yili Wang

**Affiliations:** 1 Institute for Cancer Research School of Basic Medical Science Xi’an Jiaotong University Xi’an 710061 China; 2 Department of Urology the Third Affiliated Hospital of Xi’an Jiaotong University Xi’an 710068 China; 3 Key Laboratory of Resource Biology and Biotechnology in Western China Ministry of Education Provincial Key Laboratory of Biotechnology College of Life Sciences Northwest University Xi’an 710069 China; 4 Department of Urology the First Affiliated Hospital of Xi’an Jiaotong University Xi’an 710061 China

**Keywords:** C1GalT1, O-GlcNAc modification, bladder cancer, T antigen

## Abstract

Protein glycosylation is a type of protein post-translational modification. One specific example is the modification of proteins with O-linked β-N-acetylglucosamine (O-GlcNAc) and O-linked α-N-acetylgalactosamine (O-GalNAc). Enhanced levels of both O-GalNAc and O-GlcNAc in bladder cancer (BlCa) have been reported previously. However, the interplay between O-GalNAc and O-GlcNAc has yet to be explored. Herein, we find that the expression level of core1 β-1,3-galactosyltransferase (C1GalT1), which is responsible for extending and maturing mucin-type O-glycans, is increased in BlCa. This increase is accompanied by O-GlcNAc modification of C1GalT1. This modification stabilizes C1GalT1 expression and strengthens its interaction with its chaperone Cosmc. Mutation at Thr229 or Thr233 attenuates C1GalT1 stability and facilitates its degradation via the proteasome pathway. Furthermore, a decrease in C1GalT1 inhibits the pro-tumorigenic effect on bladder cancer cells by suppressing glycolysis.

## Introduction

Protein glycosylation is a widespread post-translational modification that affects more than 70% of all human proteins [1]. This modification plays a key role in molecular recognition and cell‒cell adhesion, and its dysregulation can result in a variety of developmental defects, growth disorders, and lethal diseases. In the context of malignant transformation, abnormal protein glycosylation frequently occurs, leading to the expression of specific tumor-associated glycans [2]. Atypical glycans on cancer cells are commonly linked to tumor grade, invasiveness, and metastatic potential, and they are correlated with unfavorable overall prognoses [ 3, 4]. The linkage of glycan chains to polypeptide backbones in glycoproteins commonly occurs via the nitrogen of Asn residues (termed N-glycans) or the oxygen of Ser/Thr residues (termed O-glycans), with O-glycans further subdivided into two types: O-GlcNAcylation (O-linked β-N-acetylglucosamine, O-GlcNAc) and O-GalNAcylation (O-linked α-N-acetylgalactosamine, O-GalNAc) [5].

Polypeptide GalNActransferases (ppGalNAcTs) catalyze the addition of GalNAc to Ser/Thr to generate the Tn antigen (GalNAcα-O-Ser/Thr), which represents the initial form of O-GalNAc glycans [6]. Subsequent sequential glycosyltransferases extend and branch O-GalNAc glycans based on the Tn antigen. Core1 β-1,3-galactosyltransferase (C1GalT1, also known as T-synthase) transfers Gal to GalNAc to form Gal-β-1-3GalNAcα1-Ser/Thr (referred to as the T antigen), which serves as a precursor for the subsequent elongation and maturation of mucin-type O-glycans [7]. Truncated T and Tn antigens are prominently expressed in various types of cancers [8]. O-GlcNAc is modified by O-GlcNAc transferase (OGT), which adds O-GlcNAc, and O-GlcNAcase (OGA), which removes O-GlcNAc. However, the GlcNAc structure is typically not further modified or elongated to generate more complex structures [9]. O-GalNAc and O-GlcNAc glycosylation play crucial roles in various cellular processes, such as protein folding, localization, and degradation, as well as cellular signaling and immune responses. Moreover, anomalous O-GalNAc and O-GlcNAc glycosylation has been implicated in diverse diseases, including cancers, neurodegenerative illnesses, and autoimmune disorders [ 10, 11].

Bladder cancer (BlCa) is a frequently occurring malignancy in humans, with a fifth-place rank in terms of incidence and a continuously increasing frequency over the past decade. The non-muscle-invasive form of BlCa is prevalent in more than 70% of patients, while approximately 25% of patients are initially identified as having the muscle-invasive type. Muscle-invasive bladder cancer patients have a 50% chance of developing distant metastases and a grim prognosis [12]. Abnormal O-GalNAc glycosylation, including modifications in the T antigen and Tn antigen expression, has been reported in BlCa [ 13, 14]. Additionally, an amplified level of O-GlcNAc has also been detected in BlCa [15]. However, investigations on the interaction between O-GalNAc and O-GlcNAc in the context of BlCa have not been conducted thus far.

In a previous study, we elucidated the function of the upregulated glycosyltransferase C1GalT1 and identified the regulation of C1GalT1 via the miR-1-3p/cHP1BP3 axis at the mRNA level in BlCa [16]. In the present study, we examined the expression patterns of C1GalT1 and T antigen in BlCa cells associated with O-GlcNAc modification and deciphered the underlying mechanism of how C1GalT1 is modulated by O-GlcNAcylation.

## Materials and Methods

### Cell lines and culture

The normal bladder mucosa cells (HCV29) and bladder cancer cells (KK47 and YTS-1) were obtained from Dr. Sen-itiroh Hakomori [17]. Uroepithelia cells (SV-HUC-1), bladder cancer cells (T24, J82, RT-4 and 5637) were purchased from the Cell Bank of the Chinese Academy of Sciences (Shanghai, China). The culture media for HCV29, KK47, YTS-1, T24, RT-4, and 5637 were RPMI 1640 (Hyclone, Carlsbad, USA), while the media for J82 and SV-HUC-1 were Dulbecco’s modified Eagle’s medium (DMEM; Hyclone) supplemented with fetal bovine serum (FBS, 10%; Sigma-Aldrich, St Louis, USA) and penicillin/streptomycin (P/S, 1%; BasalMedia, Shanghai, China). All the cells were cultured in a humid and CO _2_ atmosphere (5%) at 37°C. YTS-1 cells were treated with OGT inhibitor OSMI-1 (40 μg/mL; Sigma-Aldrich) and OGA inhibitor PugNAc (20 μM; Sigma-Aldrich) for 48 h. To examine protein stability, cells were treated with the proteasome inhibitor MG132 (100 μM; MCE, Monmouth Junction, USA) and the lysosomal inhibitor chloroquine (50 μM; MCE) for 12 h, respectively.

### Total protein extraction

After being rinsed with PBS three times, all the cells were lyzed (4°C, 3 min) using RIPA buffer supplemented with protease and phosphatase inhibitors. The lysate was centrifuged (14,000 *g*, 15 min, 4°C) to collect the supernatant. The concentration of protein in the supernatant was assayed using a BCA assay kit (Beyotime, Haimen, China).

### Western blot analysis

The primary antibodies anti-C1GalT1 (#sc-100745), anti-cosmc (#sc-271829) and anti-ubiquitination (#sc-8071) antibodies were purchased from Santa Cruz Biotech (Santa Cruz, USA). The anti-GAPDH (#G9545) antibody used as control was purchased from Sigma-Aldrich. The above antibodies were diluted with TBST at 1:1000. Horseradish peroxidase (HRP)-conjugated secondary antibodies were purchased from Beyotime and diluted to 1:5000 with TBST.

The lysate supernatants were separated via SDS-PAGE. The resulting proteins were transferred to PVDF membranes (Millipore, Billerica, USA). After being blocked with skim milk (5%) in TBST buffer (20 mM Tris-HCl, 150 mM NaCl, and 0.05% Tween 20, pH 8.0) at 37°C for 2 h, the PVDF membranes were incubated with primary antibodies (4°C, overnight) and with the appropriate HRP-conjugated secondary antibodies (4°C, 2 h). The bands on the PVDF membranes were visualized with an enhanced chemiluminescence (ECL) substrate (Vazyme, Nanjing, China) and imaged on a gel documentation system (Tanon Sci & Tech, Shanghai, China) [18].

### Co-immunoprecipitation (Co-IP)

The lysate supernatant dissolved in IP lysis buffer (#P0013; Beyotime) was incubated with the primary antibody mentioned as above (4°C, 2 h) and then incubated overnight at 4°C with IP-Beads Protein A/G Plus Agarose (sc-2003; Santa Cruz). After washing with PBS three times, SDS loading buffer was added. The resulting mixture was boiled (10 min) and centrifuged (1000 *g*, 5 min), and the supernatants were analyzed by western blot analysis.

### Quantitative real-time PCR

Total RNA was extracted with Trizol reagent (CoWin Biotech, Beijing, China) and reverse transcribed with a Superscript II kit (#R232-01; Vazyme). The resulting cDNA was analyzed on RT-qPCR system, with the primers in the UltraSYBR Mixture (#Q111-02; Vazyme), as shown in Supplementary Table S1. The following system was configured as described in the instructions: 10 μL of 2× SYBR Green Mix, 0.4 μL of upstream primer (10 μM), 0.4 μL of downstream primer (10 μM), 0.8 μL of cDNA, and 8.4 μL of ddH _2_O. The reaction procedure was set as follows: 95°C for 30 s; 95°C for 5 s, 60°C for 30 s, 40 cycles; melting curve analysis: 95°C for 15 s, 60°C for 1 min, and 95°C for 15 s.

### Flow cytometry

After trypsin digestion and centrifugation (1000 *g*, 5 min), the cells were collected, washed with PBS three times, fixed with fresh paraformaldehyde (4%, 15 min, room temperature) and treated with Triton-X-100 (20 min, room temperature), followed by blocking with BSA (3%) for 30 min at room temperature. Then, the products were incubated for 1 h at 4°C with lectin labelled with a fluorophore (1:200; Beyotime) and washed with PBS three times.

### Immunofluorescence microscopy

Immunofluorescence microscopy was performed according to a previously reported protocol [18]. Briefly, cells were washed with PBS (4°C) three times, fixed with paraformaldehyde (4%, 15 min, room temperature), blocked with BSA (3%, 15 min, room temperature), and then incubated with primary antibodies (4°C, overnight). After being washed with PBS, the cells were incubated with Alexa Fluor 488 Streptavidin (#35103ES60; Yeasen, Shanghai, China) and DAPI (#28718-90-3; Sigma-Aldrich) (20 min, room temperature). A Leica confocal microscope (TCS SP8; Leica, Wetzlar, Germany) was used to obtain images.

### Plasmids and cloning

The *C1GalT1* gene was amplified from the cDNA library of YTS-1 cells, and site-specific mutagenesis was achieved via fusion PCR. The resulting *C1GalT1* gene with a site-specific mutation was cloned and inserted into the pLVX-IRES-Hgy plasmid (Takara, Shiga, Japan) and transformed into HCV29 cells [15]. Primer sequences were shown in Supplementary Table S2.

### Knockdown of *OGT* in C1GalT1

The *OGT* gene was knockdown by shRNA. shRNA vector was constructed based on pLVX-shRNA-puro (Takara). The shRNA sequence of OGT was as follow: 5′-TTTAGCACTCTGGCAATTAAA-3′.

### Wound healing assay

The cells in 6-well plates that reached confluence were treated with mitomycin (0.4 μg/mL; Sigma-Aldrich) for 30 min and then scratched with a pipette tip. After being washed with PBS, the cells were cultured in RPMI 1640 medium supplemented with mitomycin (0.04 μg/mL). Images were recorded under a microscope at 48 h [18]. The relative migration distance was calculated using Image Pro Plus software from Media (Cybernetics, Silver Spring, USA).

### Cell proliferation assay and cell cycle

After incubation with the iClick EdU solution (10 μM, 12 h; GeneCopoeia, Rockville, USA), the cells were fixed using 4% paraformaldehyde, permeabilized with 0.5% Triton X-100, and subsequently stained with EdU. The cells were rinsed with PBS before being loaded for flow cytometry analysis. For cell cycle analysis, EdU-labeled cells were treated with PI/RNase kit (Beyotime) for 30 min and subjected to flow cytometry.

### Prediction of O-GlcNAc modification and Yin Yang sites

YinOYang-1.2 ( https://services.healthtech.dtu.dk/services/YinOYang-1.2/) was used to predict the O-GlcNAc modification potential sites of the target protein. Enter the amino acid sequence of the target protein in the search bar, and when the threshold is greater than 50, it is considered that there is an O-glycosylation modification site.

### Glycoprotoemics analysis

The protein in the samples was denatured with urea (8 M) and then reduced with dithiothreitol (DTT), followed by alkylation with iodoacetamide (IAM), trypsin digestion, and purification using Oasis HLB cartridges (Waters, Milford, USA). Peanut agglutinin (PNA) lectin (Vector Laboratories, Burlingame, USA) was used for the labelling of the T-antigen moiety on peptides in the cell lysate. The protein was captured via streptavidin magnetic beads (PuriMag Biotech, Xiamen, China). Protein analysis was performed on a two-dimensional liquid chromatography-mass spectrometer (2D-LC-MS) following a previously reported protocol [16].

### Metabolism assay

The extracellular acidification rate assay (ECAR) was performed on an XF96 Extracellular Flux Analyzer (Agilen, Santa Clara, USA). Cells were plated in nonbuffered RPMI 1640 media supplemented with 25 mM glucose. Measurements were conducted under basal conditions and after the addition of 1 mM oligomycin (maximum glycolytic capacity).

### Functional enrichment analysis

The differentially expressed glycoproteins after OSMI-1 treatment were identified by >1.5-fold or <0.67-fold, *P*<0.05 as the cut-off value. GO (Gene Ontology) and KEGG (Kyoto Encyclopedia of Genes and Genomes) pathway enrichment analysis were performed using the R package “limma” and “clusterProfiler”. Biological process (BP), cellular component (CC) and molecular function (MF) are the three categories examined in GO pathway enrichment analysis. The relevant threshold of functional pathway evaluation was set as *P*<0.05.

### Statistical analysis

For statistical analysis, at least three experiments were performed. All the data are presented as the mean±standard deviation (SD). Intergroup comparisons of the data sets were performed using two-tailed Student’s *t* tests. A significance level of *P*<0.05 was used to determine statistical significance. All the statistical analyses were performed with GraphPad Prism (version 7.0; GraphPad Software; La Jolla, USA).

## Results

### Glycopattern alterations in melatonin-treated YTS-1 cells

**Figure 1:**
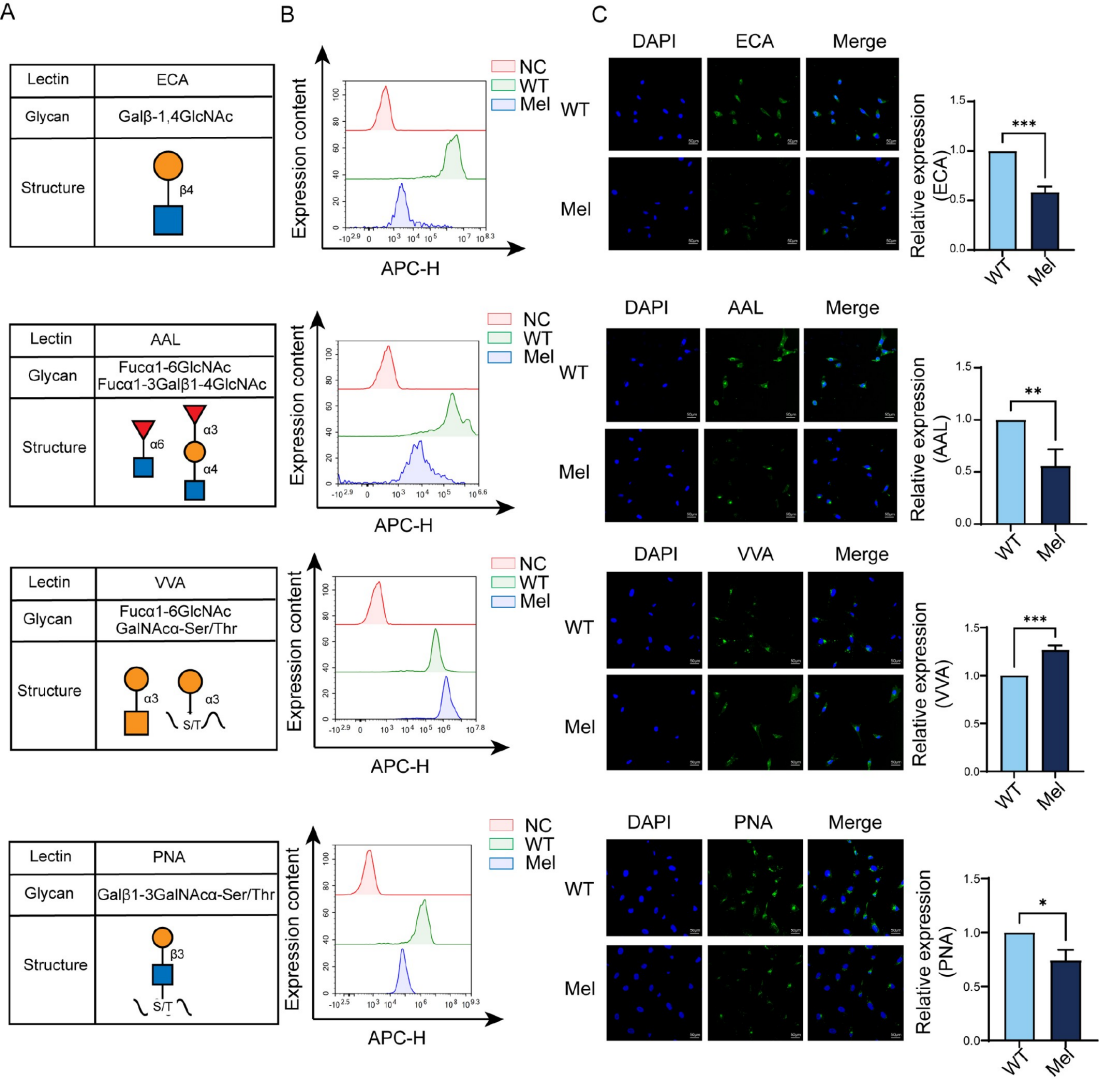
Figure 1 Changes in glycan levels in YTS-1 cells upon treatment with melatonin (A) YTS-1 cells were treated with 200 μM melatonin for 48 h and subsequently labelled with FITC-conjugated lectins (ECA, AAL, VVA and PNA). (B,C) The cells were then analyzed by flow cytometry (B) and immunofluorescence microscopy (C) to detect alterations in glycan levels. NC represents the negative control. *P<0.05, **P<0.01, ***P<0.001.

Our 2021 publication explored the impact of melatonin treatment on O-GlcNAcylation levels in bladder cancer cells. In this project, we conducted a glycopattern evaluation of overall glycans responsive to melatonin treatment using a microarray consisting of 37 lectins ( Supplementary Figure S1A). Our results revealed significant changes (>1.5-fold or <0.67-fold, *P*<0.05) in the expression of 19 different lectins, including 11 upregulated glycans and 8 downregulated glycans ( Supplementary Figure S1A). We observed 8 downregulated glycans and 11 upregulated glycans, as presented in Supplementary Figure S1B. Changes in the signals of the lectins ECA, AAL, VVA, and PNA, which specifically recognize certain glycan structures ( Figure 1A), were confirmed using lectin-based flow cytometry and immunofluorescence assays ( Figure 1B,C). The signals of ECA, AAL, and PNA decreased, while the signal of VVA increased in melatonin-treated cells, consistent with the lectin microarray results. Notably, melatonin treatment resulted in a significant downregulation of T-antigen (Gal-β-1,3-GalNAcα-Ser/Thr) expression in YTS-1 cells, as indicated by its recognition by PNA.

### Association of O-GlcNAc levels with C1GalT1 expression

**Figure 2:**
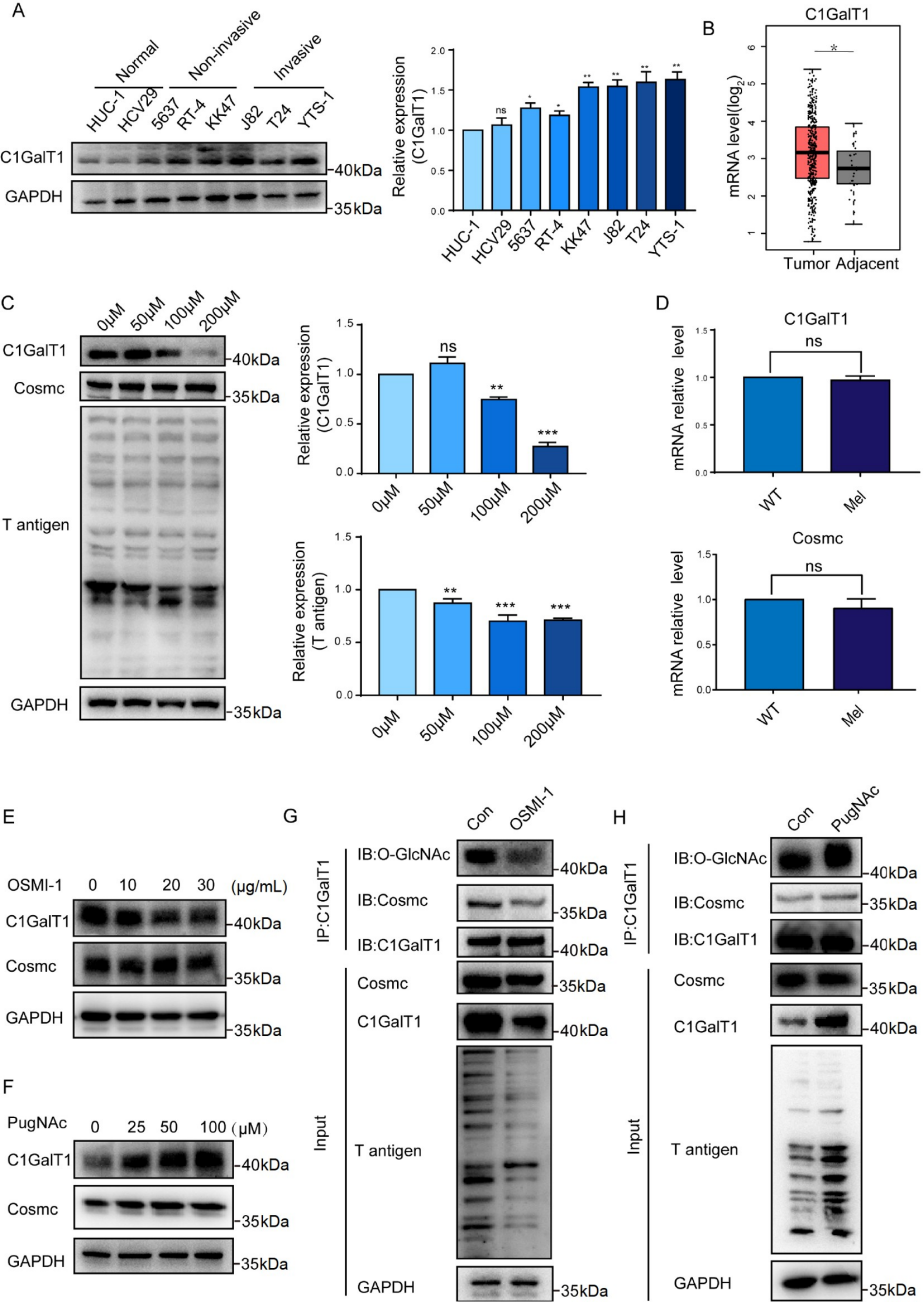
Figure 2 C1GalT1 expression and its modulation in response to melatonin treatment (A) Expression of C1GalT1 in various bladder cancer (5637, RT4, KK47, J82, T24, and YTS-1) and normal uroepithelial (HCV29 and HUC-1) cell lines, as determined by western blot analysis. (B) mRNA expression of C1GalT1 in cancer and normal tissue samples obtained from The Cancer Genome Atlas (TCGA) database. The red box represents tumor tissue, and the gray box represents adjacent tissue. (C) Expressions of C1GalT1, Cosmc, and T-antigen in YTS-1 cells treated with different concentrations of melatonin. (D) mRNA expressions of C1GalT1 and Cosmc in YTS-1 cells treated with melatonin, as analyzed by qPCR. Protein expressions of C1GalT1 and Cosmc in YTS-1 cells treated with melatonin, as determined by western blot analysis. (E) C1GalT1 and Cosmc expressions in YTS-1 cells treated with different concentrations of OSMI-1. (F) C1GalT1 and Cosmc expressions in YTS-1 cells treated with different concentrations of PugNAc. (G) Interaction between C1GalT1 and Cosmc and T-antigen levels in YTS-1 cells treated with 30 μg/mL OSMI-1 were analyzed by IP and western blot analysis. (H) Interaction between C1GalT1 and Cosmc and T-antigen levels in YTS-1 cells treated with 100 μM PugNAc were analyzed by IP and western blot analysis. *P<0.05, **P<0.01, ***P<0.001.

C1GalT1 catalyzes the transfer of Gal to Ser/Thr-GalNAc, resulting in the production of the T antigen. Analysis of bladder cancer cell lines (5637, KK47, J82, T24, and YTS-1) and normal bladder epithelial cells (HUC-1 and HCV29) revealed greater expression of C1GALT1 in bladder cancer cells than in normal cells ( Figure 2A). The TCGA database also showed enhanced C1GalT1 expression in bladder cancer tissues compared to normal tissues ( Figure 2B). Treatment of YTS-1 cells with melatonin resulted in a gradual decrease in intracellular T-antigen levels and C1GalT1 expression ( Figure 2C). Evaluation of the mRNA levels of *C1GalT1* and its molecular chaperone *Cosmc* revealed that while melatonin treatment did not significantly change *Cosmc* level, it significantly reduced *C1GalT1* level ( Figure 2D). Our previous study showed that melatonin can reduce O-GlcNAcylation levels. Treatment of YTS-1 cells with the OGT inhibitor OSMI-1 resulted in a decrease in C1GalT1 level but not Cosmc level ( Figure 2E). Conversely, treatment with the OGA inhibitor PugNAc caused a gradual increase in C1GalT1 expression ( Figure 2F). The O-GlcNAcylation of C1GalT1 and the Cosmc/C1GalT1 interaction were reduced in OSMI-1-treated YTS-1 cells ( Figure 2G) but increased in PugNAc-treated cells ( Figure 2H). The same results were obtained in both YTS-1 cells overexpressing OGT and YTS-1 cells with silenced *OGT* ( Supplementary Figure S2A,B). These results demonstrate the strong effect of O-GlcNAcylation on C1GalT1 stability and its interaction with Cosmc.

### The impact of O-GlcNAc on C1GalT1 stability

**Figure 3:**
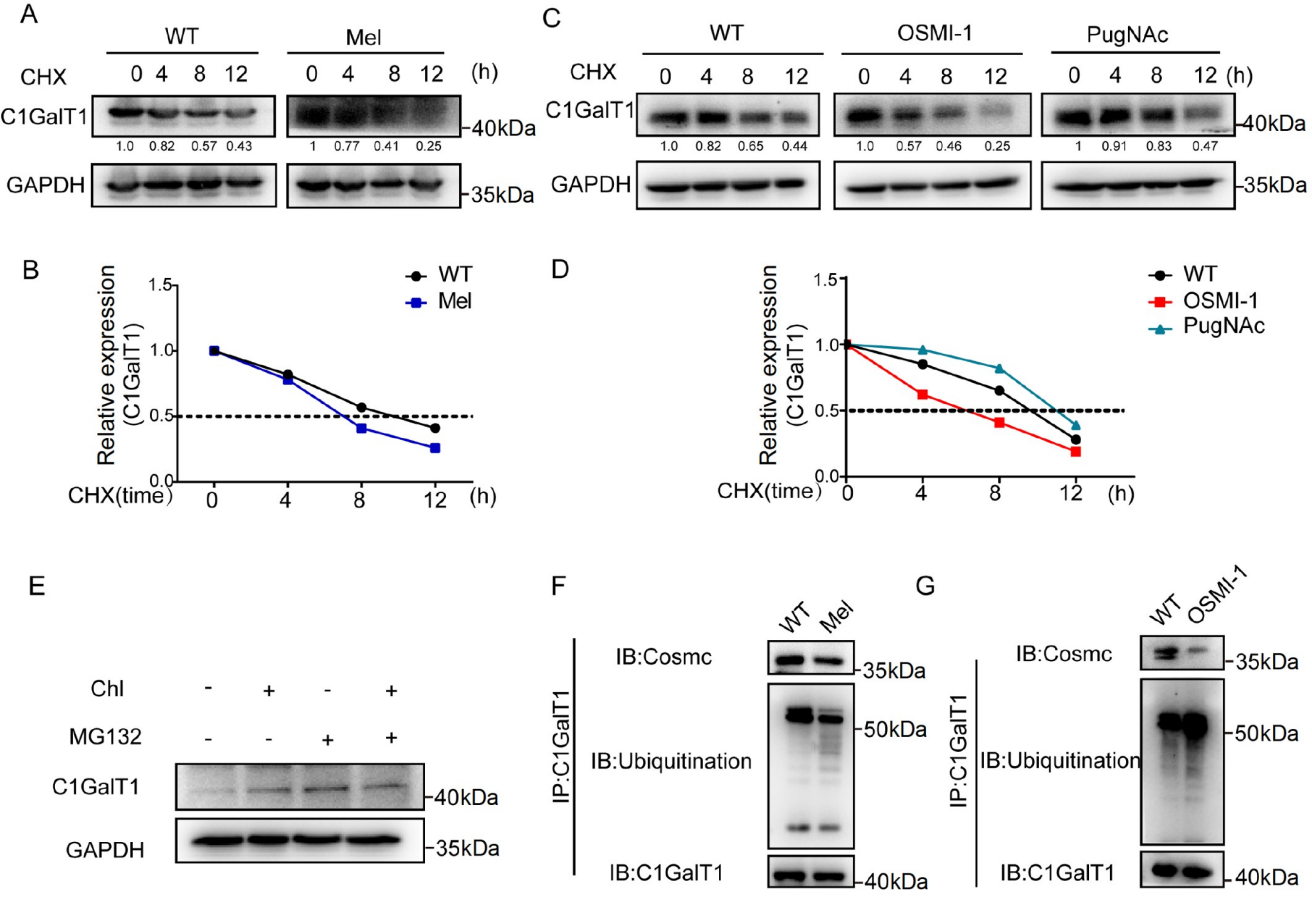
Figure 3 Effect of O-GlcNAcylation on C1GalT1 stability (A) Expression of C1GalT1 in YTS-1 cells treated with melatonin and in untreated cells treated with CHX at various time points. (B) Half-life of C1GalT1 in YTS-1 cells treated with melatonin. (C) Expression of C1GalT1 in YTS-1 cells treated with OSMI-1 or PugNAc and in untreated cells treated with CHX at various time points. (D) Half-life of C1GalT1 in YTS-1 cells treated with OSMI-1 or PugNAc. (E) Expression of C1GalT1 in YTS-1 cells treated with MG132 or chloroquine. (F) Ubiquitination level of C1GalT1 and the interaction between C1GalT1 and Cosmc in YTS-1 cells treated with 200 μM melatonin. (G) Ubiquitination level of C1GalT1 and the interaction between C1GalT1 and Cosmc in YTS-1 cells treated with 30 μg/mL OSMI-1.

It is known that O-GlcNAc modification plays key roles in protein stability [19]. Here, we investigated whether the stability of C1GalT1 is impacted by O-GlcNAc modification. Specifically, YTS-1 cells were subjected to melatonin treatment, which was followed by CHX administration to halt protein synthesis. In YTS-1 cells treated with melatonin, the half-life of C1GalT1 was reduced ( Figure 3A,B). YTS-1 cells were treated with OSMI-1 and PugNAc to inhibit OGT and OGA, followed by CHX treatment. The half-life of C1GalT1 was decreased by OSMI-1 but increased by PugNAc ( Figure 3C,D). When YTS-1 cells were treated with MG132 (an inhibitor of the proteasome) or chloroquine (an inhibitor of lysosomes), C1GalT1 degradation occurred via the proteasome pathway ( Figure 3E). Melatonin or OSMI-1 treatment of YTS-1 cells caused significantly increased ubiquitination ( Figure 3F,G). Our results indicate that a reduction in O-GlcNAcylation of C1GalT1 is associated with increased ubiquitination and degradation of the protein.

### The key O-GlcNAc sites on C1GalT1

**Figure 4:**
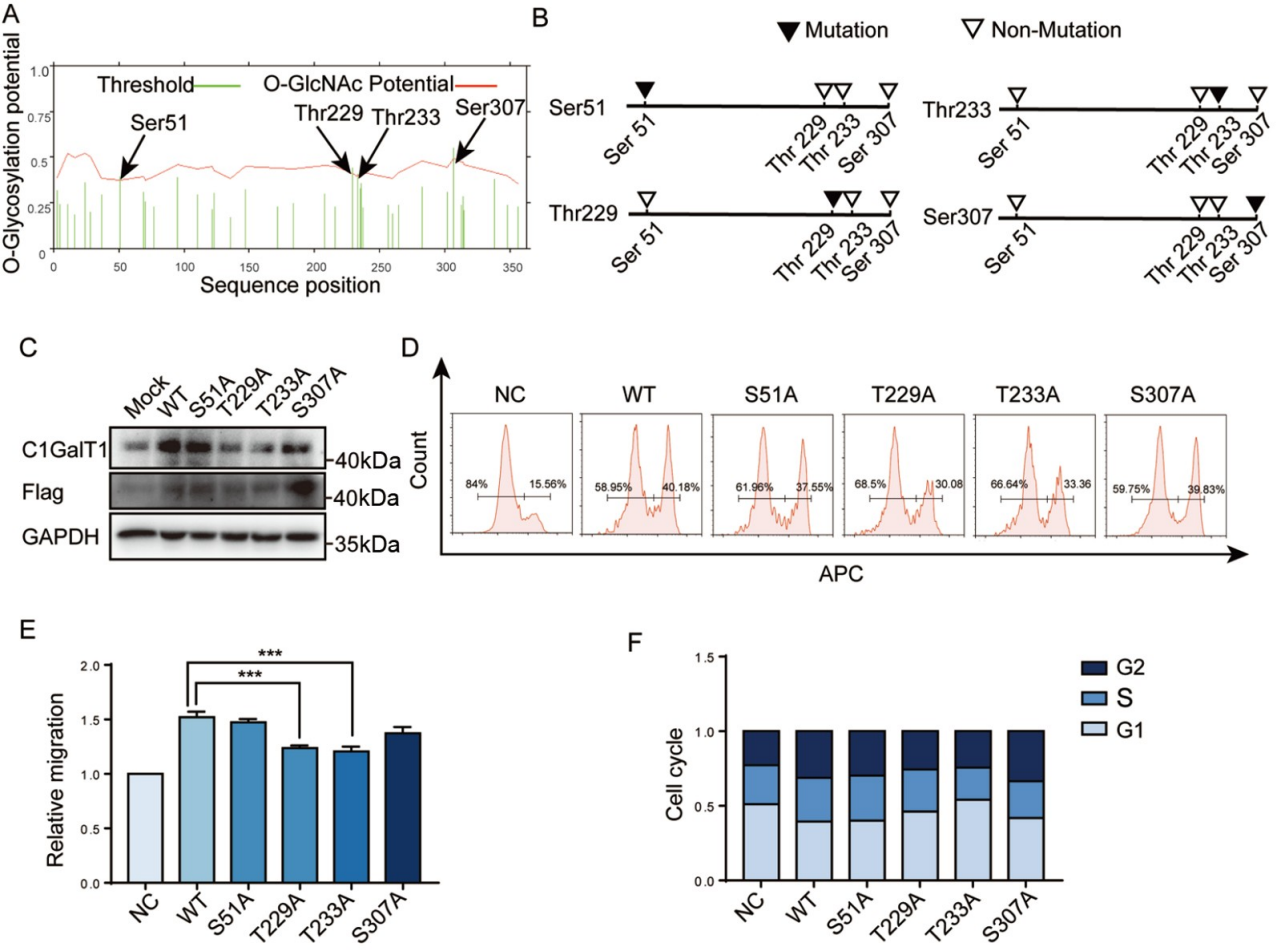
Figure 4 O-GlcNAcylation sites on C1GalT1 (A) Prediction of key O-GlcNAc sites on C1GalT1. (B) Potential O-GlcNAcylation sites (black triangles) and point mutations (white triangles) on C1GalT1. (C) Expressions of C1GalT1 and Flag in various C1GalT1 mutant HCV29 cells. (D) Proliferation of various C1GalT1 mutant HCV29 cells. (E) Cell migration of various C1GalT1 mutant HCV29 cells. (F) Cell cycle distribution of various C1GalT1 mutant HCV29 cells. ***P<0.001.

The prediction results from YinOYang 1.2 indicated the presence of four potential O-GlcNAc modification sites: Ser51, Thr229, Thr233, and Ser307 ( Figure 4A). Moreover, HCV29 cell lines expressing different C1GalT1 mutants in which Ser51, Thr229, Thr233, and Ser307 were replaced by Ala were constructed ( Figure 4B). T229A and T233A mutant cells presented the lowest protein levels of C1GalT1 ( Figure 4C and Supplementary Figure S3A) but not the lowest mRNA levels ( Supplementary Figure S3B). The half-life of C1GalT1 decreased in the T229A and T233A strains ( Supplementary Figure S3C). Furthermore, T229A and T233A presented a significant reduction in cell proliferation ( Figure 4D) and migration ( Figure 4E and Supplementary Figure S4A), and a greater proportion of cells arrested at the G1 phase ( Figure 4F and Supplementary Figure S4B). Our findings suggest that O-GlcNAc modification sites, especially T229A and T233A, play a critical role in maintaining the stability of the C1GalT1 protein.

### Identification of T antigen-bearing target glycoproteins

**Figure 5:**
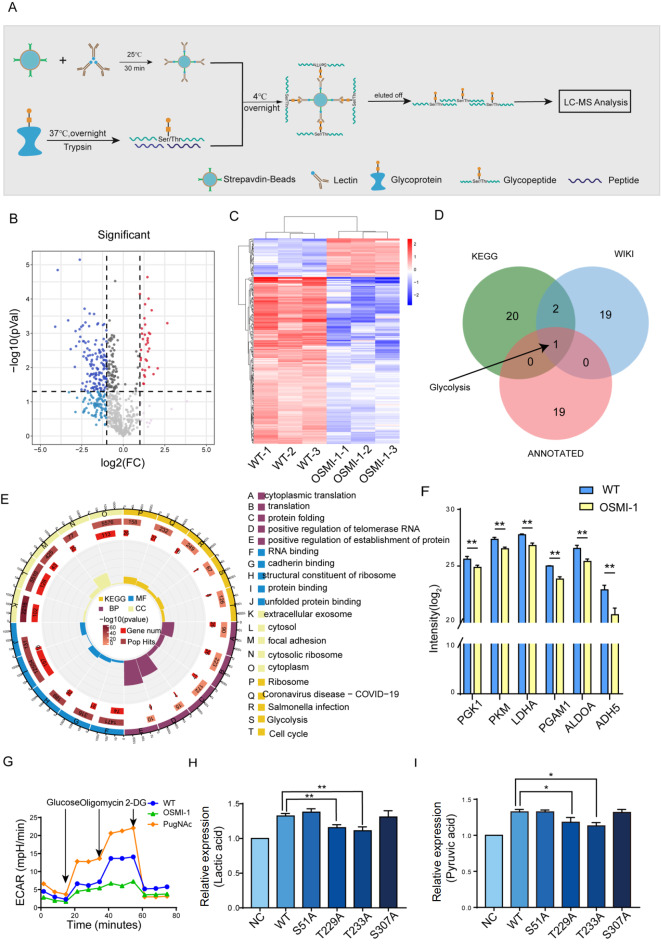
Figure 5 Differentially expressed glycoproteins with T-antigen (A) Workflow of glycoproteomic analysis. (B) Volcano plot showing the differentially expressed proteins identified in OSMI-1-treated and untreated YTS-1 cells. The log10 P value is plotted against the log2-fold change (treatment vs control). (C) Heatmap displaying the differentially expressed proteins in OSMI-1-treated YTS-1 cells compared to untreated YTS-1 cells. Red represents upregulation, while blue represents downregulation. (D) Venn diagram illustrating the number of differentially expressed proteins identified in the KEGG, Wiki, and annotated pathways. (E) GO enrichment analysis of differentially expressed glycoproteins with T-antigens. (F) Expressions of glycoproteins enriched in the glycolysis pathway and the expressions of various C1GalT1 mutants in HCV29 cells. (G) The extent of extracellular acidification in YTS-1 cells treated with OSMI-1 or PugNAc was measured by a Seahorse system. (H) Lactic acid levels in HCV29 cells. (I) Pyruvic acid levels in HCV29 cells. *P<0.05, **P<0.01.

To identify T antigen-bearing target glycoproteins, we identified glycopeptides labelled with lectin-streptavidin ( Figure 5A). Proteomic analysis revealed the expression patterns of glycoproteins in response to OSMI-1 induction. A total of 208 glycoproteins demonstrated differential expression, as represented in a volcano plot ( Figure 5B). Among them, 39 glycoproteins were upregulated, and 169 glycoproteins were downregulated in OSMI-1-treated YTS-1 cells ( Figure 5C). KEGG analysis revealed that these proteins were enriched mainly in the glycolysis pathway ( Figure 5D). GO analysis revealed that T-antigen-modified proteins were involved in the biological process of cytoplasmic translation, the cellular component of extracellular exosome, and the molecular function of identical protein binding ( Figure 5E). Notably, OSMI-1 treatment of YTS-1 cells significantly reduced the levels of PGK1, PKM, LDHA, PGAM1, ALDOA, and ADH5, which are involved in the glycolysis pathway ( Figure 5F). O-GlcNAc modification of PGK1, PKM1, LDHA, PGAM1, ALDOA and ADH5 was observed in YTS-1 ( Supplementary Figure S5A). Protein‒protein interaction network analysis revealed that the target proteins formed clusters that were enriched in glycolysis ( Supplementary Figure S5B). Therefore, we evaluated the level of glycolysis by measuring the ECAR, an indicator of aerobic glycolysis, the process by which glucose is converted to lactate in the presence of oxygen. The ECAR decreased in YTS-1 cells treated with OSMI-1 but increased in YTS-1 cells treated with PugNAc ( Figure 5G). These results indicated that O-GlcNAc plays a role in the regulation of glycolysis.

In addition, we detected the levels of lactic acid and pyruvic acid, which are involved in glycolysis. The T229A and T233A cells presented the lowest levels of lactic acid and pyruvic acid ( Figure 5H,I). To investigate whether the O-GlcNAc modification of C1GalT1 promotes cell proliferation and migration through enhanced glycolysis, we measured cell proliferation and migration in T229A and T233A cells treated with 2-deoxyglucose (2-DG), a compound that mimics D-glucose and inhibits glycolysis. The proliferation and migration abilities were decreased in YTS-1 cells treated with 2-DG, but these decreases were diminished in T229A and T233A cells ( Supplementary Figure S5C,D). In conclusion, our findings suggest that Thr229 and Thr233 are important O-GlcNAc sites of C1GalT1 and that decreased O-GlcNAc levels in C1GalT1 could decrease glycolysis level by influencing glycoproteins modified by T-antigens.

## Discussion

C1GalT1 is a pivotal glycosyltransferase involved in the biosynthesis of O-linked mucin-type glycans on glycoproteins [2], and there is mounting evidence suggesting that C1GalT1 is upregulated in a variety of epithelial-origin cancers, including breast, colon, esophageal, gastric, head and neck, hepatocellular, pancreatic, and prostate cancers [20]. Our study demonstrated that C1GalT1 expression is markedly increased in invasive bladder cancer cells compared to noninvasive bladder cancer cells and normal bladder cells. Moreover, our previous investigation indicated that O-GlcNAcylation, which occurs predominantly in cytoplasmic proteins, is substantially elevated in bladder cancer cells [15]. Therefore, we hypothesize that there is a potential correlation between C1GalT1 and O-GlcNAcylation.

O-GlcNAcylation is a distinctive posttranslational modification that involves the attachment of a carbohydrate to the hydroxyl group of a Ser/Thr residue [20]. Recent studies have emphasized the pivotal role of O-GlcNAcylation in the maintenance of protein stability [21]. It has been reported that O-GlcNAc modification acts as an intrinsically occurring inhibitor of the proteasome system and that O-GlcNAc glycosylation is reduced upon ubiquitination [22]. Our research revealed that an increase in the ubiquitination of C1GalT1 correlates with a reduction in O-GlcNAcylation, which eventually leads to C1GalT1 degradation through the proteasome pathway. The activation of C1GalT1 enzyme activity depends on the presence and function of Cosmc, a molecular chaperone located in the endoplasmic reticulum (ER) [23]. In the absence of Cosmc, incorrectly folded C1GalT1 peptides tend to aggregate due to interactions with other molecular chaperones, ultimately resulting in their degradation by the proteasome system [ 24, 25]. In agreement with our findings, we observed a decrease in the interaction between Cosmc and C1GalT1, as well as a decrease in the O-GlcNAcylation of C1GalT1. Our team also identified four potential O-GlcNAc sites in C1GalT1, namely, Ser51, Thr229, Thr233, and Ser307. Mutations in O-GlcNAcylation at T229A and T233A resulted in changes in protein stability and cellular behavior, inducing alterations in the cell cycle, migration, and proliferation in bladder cancer cells. These results suggest that T229A and T233A are essential for maintaining the stability and function of C1GalT1.

The T antigen is a glycan epitope produced by C1GalT1 that is highly prevalent in bladder cancer cells but rarely expressed in normal bladder cells [26]. Alterations in C1GalT1 expression can lead to variations in the glycosylation of a wide range of cell membrane glycoproteins, including mucin proteins, growth factor receptors, adhesion molecules, and death receptors [ 27, 28]. These modifications can potentially lead to changes in the interactions between these cell surface molecules and their respective binding ligands, which can ultimately lead to changes in cancer cell behavior and activity [29]. Our investigation revealed that T antigen modification occurred in glycolysis-related proteins, such as PGK1, PKM, LDHA, PGAM1, and ALDOA. Moreover, as T antigen levels decreased, the expressions of these glycolysis-related proteins also decreased, indicating that C1GalT1 plays a functional role in regulating the glycolysis system by modulating T antigen levels. Reduced O-GlcNAcylation of C1GalT1 stimulates its degradation, leading to a decrease in T antigen levels and subsequent downregulation of glycolysis in cells. This reduction inhibits the metabolic conversion of glucose to UDP-GlcNAc, which serves as a substrate for O-GlcNAcylation [30], by inhibiting glycolysis-related proteins that are modified by the T antigen. This regulatory process inhibits the pro-tumorigenic effect of C1GalT1 on bladder cancer and establishes a beneficial feedback loop.

## Supporting information

23630Supplementary_Table_4

23630Supplementary_Table_3

23630Supplementary_material_0725

## Supplementary Data

Supplementary data is available at *Acta Biochimica et Biophysica Sinica* online.
