# Supplementary material for: O-GlcNAcylation determines the function of the key O-GalNAc glycosyltransferase C1GalT1 in bladder cancer: O-GlcNAcylation governs pro-tumorigenic role of C1GalT1 in bladder cancer
Source: Acta Biochim Biophys Sin (Shanghai). 2024 Aug 8;56(8):1108–17. doi: 10.3724/abbs.2024129 (PMC11399441; doi:10.3724/abbs.2024129)
Supplement: 23630Supplementary_material_0725 [file 23630Supplementary_material_0725.pdf]

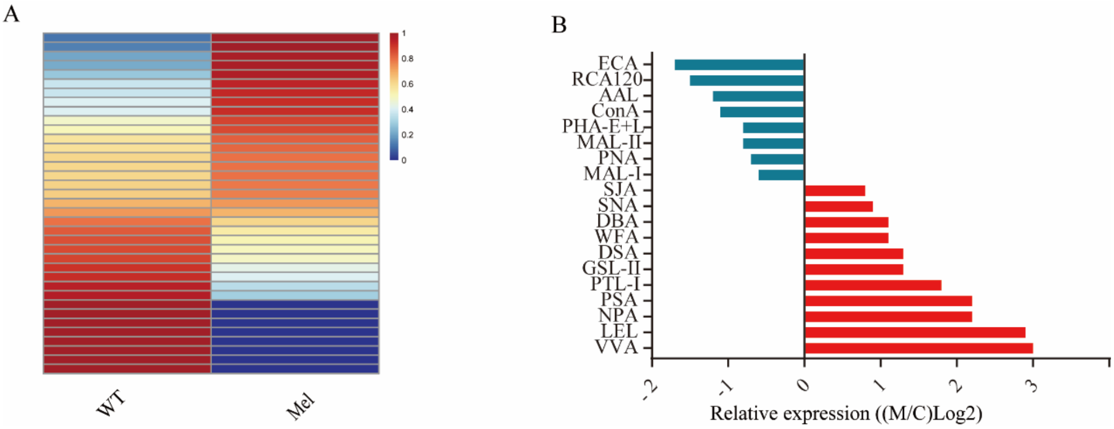

Supplementary Figure S1. The differential glycan profiles in melatonin-treated and nontreated cells (A) Heatmap of differentially expressed glycans in melatonin-treated and nontreated YTS-1 cells. Red, upregulation. Blue, downregulation. (B) Histogram of identified differentially glycan expression use the cutoff of fold change >1.5, or < 0.67 and *P*-value > 0.05 in melatonin-treated and nontreated YTS-1 cells.

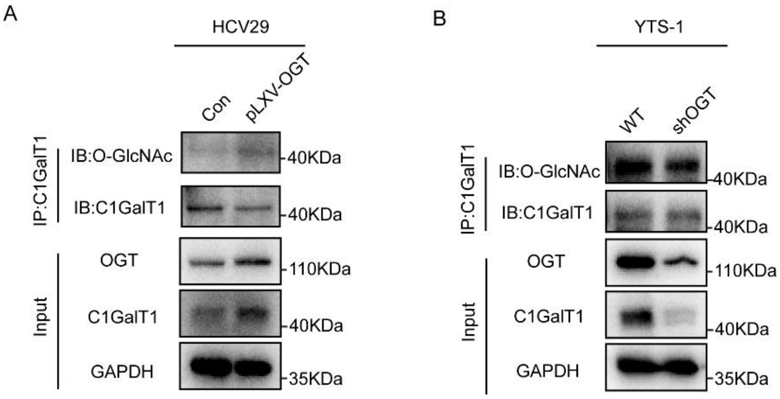

Supplementary Figure S2 The influence of O-GlcNAc modification on the expression of C1GalT1 (A) O-GlcNAc level of C1GalT1 in HCV29 overexpressing OGT. (B) O-GlcNAc level of C1GalT1 in YTS-1 silencing OGT.

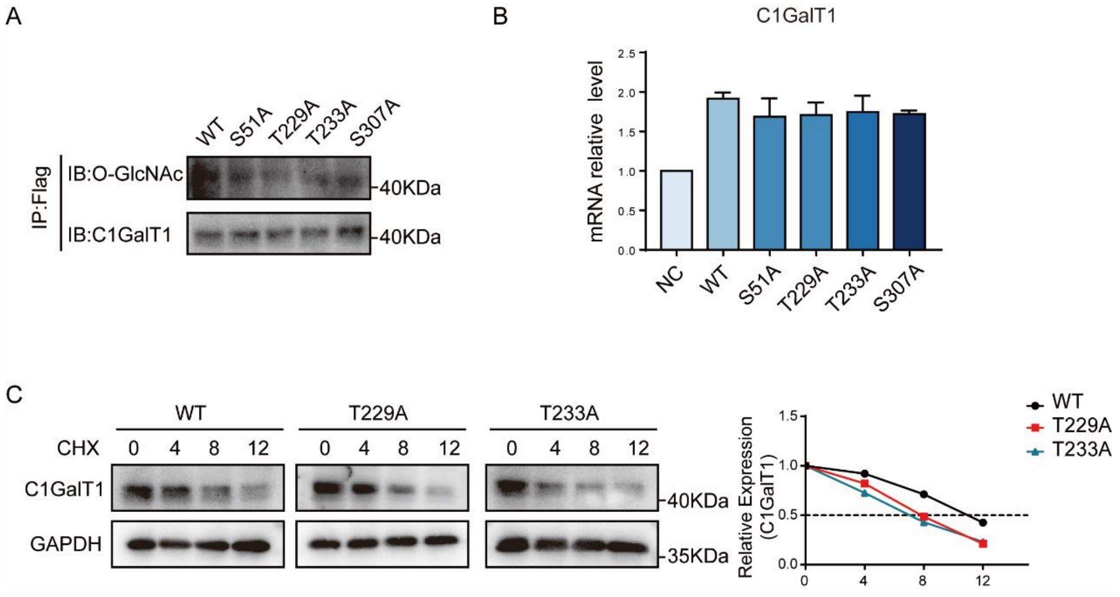

Supplementary Figure S3. The influence of various C1GalT1 mutants on C1GalT1 expression (A) O-GlcNAc level of C1GalT1 in HCV29 expressing various C1GalT1mutants. (B) C1GalT1 expression at mRNA level in C1GalT1 mutant cells. (C) The half-life of C1GalT1 in HCV29 expressing various C1GalT1 mutants.

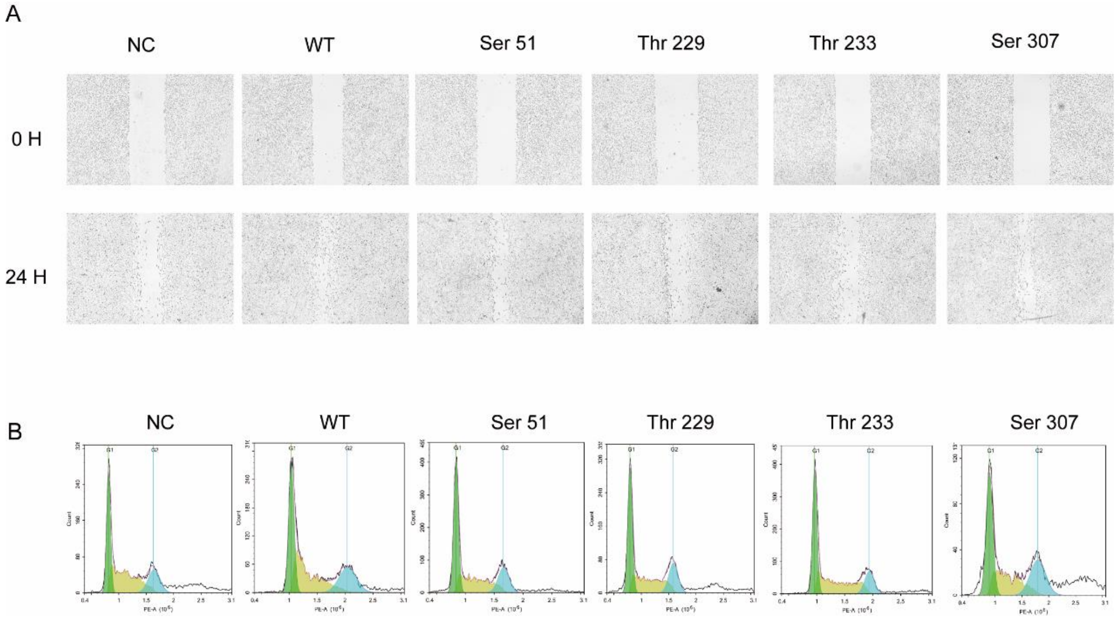

Supplementary Figure S4 The influence of various C1GalT1 mutants on cell migration and cell cycle (A) Wound assay of C1GalT1 mutant cells. (B) Cell cycle of various C1GalT1 mutants in HCV29 cells.

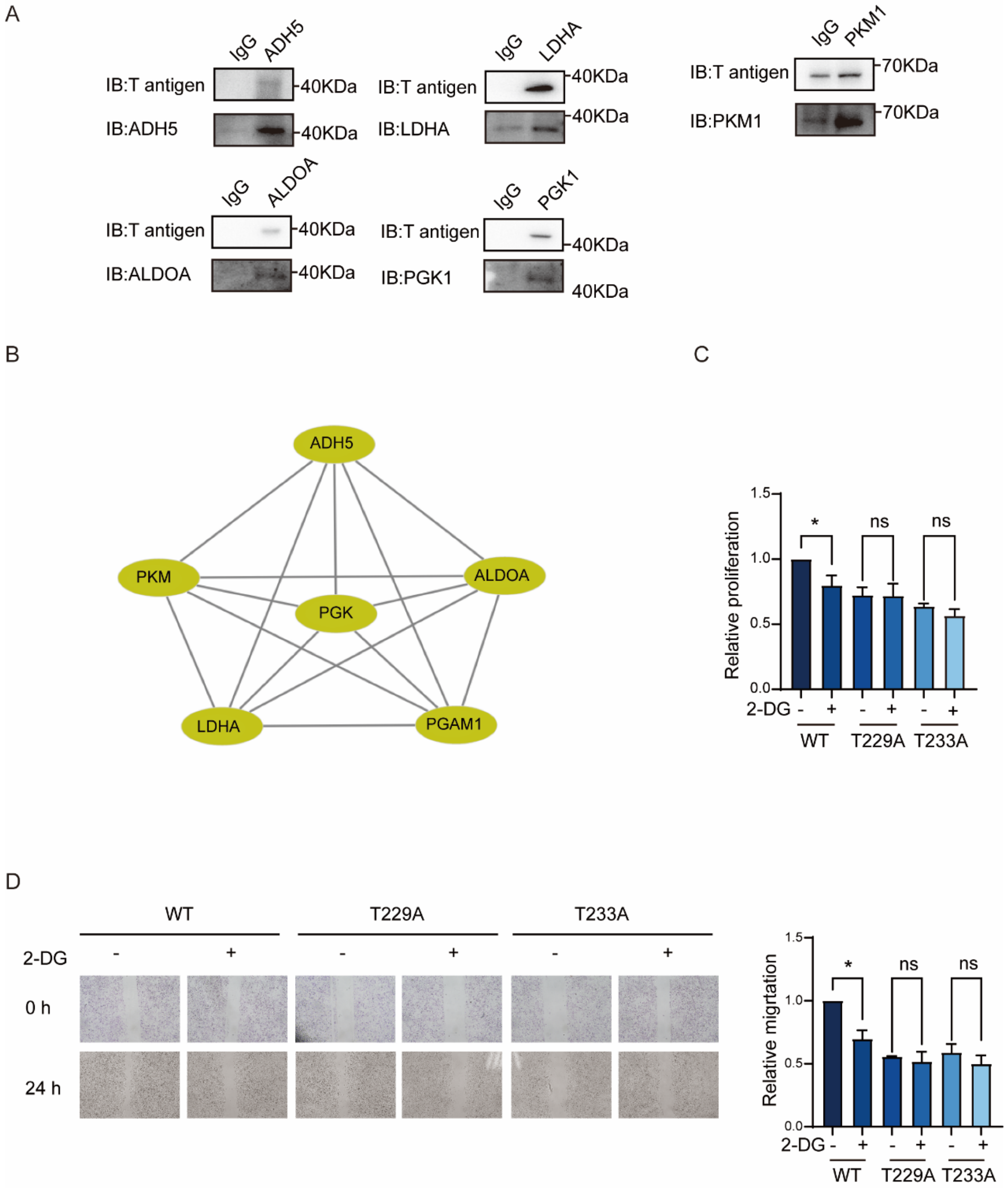

Supplementary Figure S5. Glycolysis ability was regulated by T antigen (A) O-GlcNAc level of ADH5, LDHA, PKM1, ALDOA and PGK1 in YTS-1 cell. (B) Protein protein interaction network of glycoproteins with T-antigen in glycolysis pathway. (C) Ability of proliferation in T229A and T233A treated with 5 mM 2-DG by CCK8. (D) Migration in T229A and T233A treated with 5 mM 2-DG by wound assay.

Supplementary Table S1. Sequence of primers for RT-qPCR analysis

Primer name	Target gene	Primer sequence (5′→3′)
C1GalT1-F	*C1GalT1*	TCCTCTGTGGATCAGCAATAGG
C1GalT1-R		TTAGGCTGGGTGTCAACCTTT
Cosmc -F	*Cosmc*	AGTTTGCCTGAAATATGCTGGA
Cosmc-R		GGGGTGATAAGTCATTGCCTCT
β-actin-F	*β-actin*	CTCCATCCTGGCCTCGCTGT
β-actin-R		GCTGTCACCTTCACCGTTCC

Supplementary Table S2. Sequence of primers for the establishment of plasmids and cloning

Primer name	Description	Primer sequence (5′→3′)
C1GalT1-F	Full-length C1GalT1	CCGCTCGAGATGGCCTCTAAATCCTGGCTGAAT
C1GalT1-R		GTAGGATTTCCTAACTTCACTTTTGTATCTTCATTTTTGT
Ser 51-F	Ser51 (C1GalT1)	CTCATGCAAGGCATGCAGATGAT
Ser 51-R		ATCATCTGCATGCCTTGCATGAG
Thr 229-F	Thr229 (C1GalT1)	TGCATTTAAAGCAGACAAGTGTACAC
Thr 229-R		GTGTACACTTGTCTGCTTTAAATGCA
Thr 233-F	Thr233 (C1GalT1)	GACAAGTGTGCACATAGTTCCTCC
Thr 233-R		GGAGGAACTATGTGCACACTTGTC
Ser 307-F	Ser307 (C1GalT1)	ATCTTGCAGTTGCTTTTCACTATGTT
Ser 307-R		AACATAGTGAAAAGCAACTGCAAGAT
